# The Swedish system for compensation of patient injuries

**DOI:** 10.3109/03009730903350749

**Published:** 2010-04-07

**Authors:** Henry Johansson

**Affiliations:** Department of Surgical Sciences, University Hospital, UppsalaSweden

**Keywords:** Health care-related injuries, patient injury compensation, the Swedish system

## Abstract

Since 1975 Sweden has had a patient insurance system to compensate patients for health-related injuries. The system was initially based on a voluntary patient insurance solution, but in 1997 it was replaced by the Patient Insurance Act. The current Act covers both physical and mental injuries. Although about 9,000–10,000 cases are processed in Sweden annually, compensation is paid in barely half of these cases. In the Swedish patient injury claim processing system, the Patient Claims Panel is the authority that plays an important role in ensuring fair and consistent application of the Act.

## Introduction

International studies have shown that the incidence of adverse events varies between 2.9% (1) and 16.6% (2) among hospital patients. These varying figures are explained in part by the difficulty in defining the concept of adverse event. However, data indicate that about half of the events are considered preventable, and about 5% contribute to death. Although extrapolation is difficult, international figures suggest that about 30,000 patients in Sweden (for a population of about 9 million) are treated annually for avoidable patient injuries and, for 1,500 individuals, these preventable events lead to death. These figures are in sharp contrast to the official statistics reporting 9,000–10,000 patient injuries per year (3). The data indicate that about 0.2% all admissions in Sweden result in a patient injury claim.

## The Swedish patient insurance system

Sweden has had a patient insurance system to compensate patients for health-related injuries since 1975. This system was initially based on a voluntary patient insurance solution, but in 1997 it was replaced by the Patient Insurance Act. The law contains provisions regarding the right to injury compensation and the duty of the care provider to carry patient insurance that covers compensation for injuries. In Sweden the county councils are responsible for most medical services and are therefore the target of most compensation claims. They use a common insurer, Landstingens Ömsesidiga Försäkringsbolag (LÖF) (The County Council's Mutual Insurance Company), which in turn commissions the company Personskadereglering AB (PSR) to process the compensation claims. PSR processes more than 90% of all patient injury claims.

The Act, which covers both physical and mental injuries, requires that a causal relationship must be established between injury and medical care and that it can be demonstrated that injury could have been avoided. The main types of compensable injuries are diagnostic, treatment, and infection-related injuries.

## The role of the Patient Claims Panel

The Patient Claims Panel is part of the Swedish patient injury system. The committee endeavours to achieve uniformity in practice and issues advisory opinions in compensation cases referred to the panel by the patient, care provider, or insurer. The panel consists of a chairman, who is or has been a professional judge, and six members. Three of these members represent the interest of patients, and one is a medical expert, one is familiar with the insurers' personal injury settlement process, and one is an expert in the Swedish health care system.

In 2004 the Patient Claims Panel considered over 1,000 cases to decide whether it was justified to change previous decisions (4). Almost all cases were referred to the panel by PSR. In 11% of these cases the panel advised changing of the previous assessment to the patient's advantage. Most cases involved diagnostic and treatment injuries. With respect to diagnostic injuries, the panel felt that in some cases the injury was caused by an incorrect diagnosis or that the diagnosis was delayed because the symptoms were misunderstood or interpreted in a manner that deviated from a normal standard. The committee felt that the diagnostic injury had caused an incorrect or delayed treatment. According to the committee, the treatment injuries were such that injury could have been avoided by choosing a different procedure, or by choosing a different, less risky therapy. In some cases additional compensation was justified to cover economic loss or to provide reasonable compensation for pain and suffering. In a few cases the claim was not considered statute-barred.

PSR processes about 9,000 claims annually, and experience has shown that about 45% of the claimants receive compensation. The majority of compensation is given for physical injuries, very few for mental cases. During 1997–2006 PSR processed 965 mental cases, and of them only 245 received compensation, i.e. 25% (5). However, the figures suggest that even in Sweden, about half of all patient injuries are considered avoidable. With respect to diagnostic injuries it is important to emphasize that a delayed diagnosis does not necessarily influence treatment strategies, but cancer patients may face a worse prognosis. For the afflicted patient this can mean mental suffering, and such patients usually receive compensation for indirect damages. In several of the cases in which the panel, unlike PSR, considered a treatment injury to be present, PSR did not apply retroactive logic, suggesting a fundamental difference in the interpretation of this paragraph of the law.

A retroactive assessment is based on consideration of whether the injury could have been avoided, purely hypothetically, with knowledge of treatment outcome. From a strictly medical view-point, the patient may have received the right treatment, even though the patient, according to retroactive assessment, is entitled to compensation for this injury. The panel's experience is that retroactive logic has been particularly difficult to apply to birth injuries. Retroactive logic requires consideration of whether the injury could have been avoided, either through a different treatment method or through another way of administration of the chosen form of therapy, provided that the treatment and administration had simultaneously been less risky for the patient. In other words, retroactive determination requires simultaneous consideration of the medico-legal aspects in order to determine whether the injured patient is entitled to compensation.

## Patient safety in health care

Patient safety is an important issue in health care that must be considered at all times. The Swedish Patient Injury Act protects the best interests of the patients so that people who suffer ‘avoidable injuries’ can receive compensation–and do so without incurring legal expenses. Another advantage of the Patient Injury Act is that it is completely free of punishment and is not associated in any way with the Swedish Medical Responsibility Board, where ‘incorrect’ treatment may result in disciplinary consequences for the person involved in the form of an admonition or warning. With the Patient Injury Act, the right to compensation is determined based on objective grounds, in accordance with the provisions of the Act and the organization which evaluates patient injuries. The Patient Claims Panel plays a crucial role here as a ‘superior instance’ to provide advisory statements in cases brought before the panel by patients or other injured parties, care providers, insurers, or courts and tribunals. Although the panel's opinions are advisory, in principle the insurer always complies with them. In about 10% of cases the panel reaches a conclusion that differs from that of the insurer. The panel's important duty is to ensure fair and consistent application of the Patient Injury Act.

## Conclusion

A key goal for health care is to ensure good quality and minimize the risk of errors. In Sweden, as in many other countries, we are trying to create a safer health care system, partly through the increasing use of incident reports and various types of quality databases. At the same time that Sweden's adverse event reporting system is becoming more efficient, it is important that doctors and other health care workers are aware of patients' options for compensation when an injury occurs.
